# Head and neck tumors angiogenesis imaging with ^68^Ga-NODAGA-RGD in comparison to ^18^F-FDG PET/CT: a pilot study

**DOI:** 10.1186/s13550-020-00638-w

**Published:** 2020-05-07

**Authors:** Steve Durante, Vincent Dunet, François Gorostidi, Periklis Mitsakis, Niklaus Schaefer, Judith Delage, John O. Prior

**Affiliations:** 1 Department of Diagnostic and Interventional Radiology, Lausanne University Hospital and University of Lausanne, Rue du Bugnon, 46 Lausanne, Switzerland; 2 Department of Nuclear Medicine and Molecular Imaging, Lausanne University Hospital and University of Lausanne, Lausanne, Switzerland; 3 Department of Otolaryngology, Head and Neck Surgery, Lausanne University Hospital and University of Lausanne, Lausanne, Switzerland; 4 Department Pharmacy, Unit of Radiopharmacy, Lausanne University Hospital, Lausanne, Switzerland

**Keywords:** Head and neck cancer, PET, ^18^F-FDG, ^68^Ga-NODAGA-RGD, Angiogenesis

## Abstract

**Background:**

Angiogenesis plays an important role in head and neck squamous cell carcinoma (HNSCC) progression. This pilot study was designed to compare the distribution of ^68^Ga-NODAGA-RGD PET/CT for imaging α_v_β_3_ integrins involved in tumor angiogenesis to ^18^F-FDG PET/CT in patients with HNSCC.

**Material and methods:**

Ten patients (aged 58.4 ± 8.3 years [range, 44–73 years], 6 males, 4 females) with a total of 11 HNSCC were prospectively enrolled. Activity mapping and standard uptake values (SUV) from both ^68^Ga-NODAGA-RGD and ^18^F-FDG PET/CT scans were recorded for primary tumor and compared with the Wilcoxon signed-rank test. The relation between the SUV of both tracers was assessed using the Spearman correlation.

**Results:**

All HNSCC tumors were visible with both tracers. Quantitative analysis showed higher ^18^F-FDG SUV_max_ in comparison to ^68^Ga-NODAGA-RGD (14.0 ± 6.1 versus 3.9 ± 1.1 g/mL, *p* = 0.0017) and SUV_mean_ (8.2 ± 3.1 versus 2.0 ± 0.8 g/mL, *p* = 0.0017). Both ^18^F-FDG and ^68^Ga-NODAGA-RGD uptakes were neither correlated with grade, HPV status nor p16 protein expression (*p* ≥ 0.17).

**Conclusion:**

All HNSCC tumors were detected with both tracers with higher uptake with ^18^F-FDG, however. ^68^Ga-NODAGA-RGD has a different spatial distribution than ^18^F-FDG bringing different tumor information.

**Trial registration:**

NCT, NCT02666547. Registered 12.8.2012.

## Background

Cancer is the second cause of mortality and morbidity in industrial countries and is expected to become even more predominant in the future. Head and neck tumors are frequent and represent in Switzerland an incidence of roughly 1000 new cases annually. Around 70% of them are diagnosed in advanced stages with a 5-year survival rate of 50% [1, 2]. Excessive alcohol consumption and smoking are commonly encountered in most head and neck squamous cell carcinoma (HNSCC) patients aged 55 years and older. In the last 10 years, the incidence of HNSCC in Western countries has increased due to rising incidence of human papillomavirus (HPV)-associated SCC. In this category, patients are younger at diagnosis, with increasing numbers under the age of 40 [3].

^18^F-FDG PET/CT has demonstrated good sensitivity and specificity of around 80–100% in staging and following-up HNSCC [4–7], with no difference between HPV positive and negative. Angiogenesis plays a crucial role in tumor growth as well as in treatment resistance [3, 8] and represents an important target for the treatment of solid tumors with different expression of integrins on tumoral vessels in comparison with normal vessels [8–12]. Novel angiogenesis-targeting therapies have been developed with good response alone or in combination with conventional chemoradiotherapies [13, 14]. Morphologic imaging like MRI can only indirectly show angiogenesis with injection of gadolinated contrast, but it is limited by procedure time, lack of sensitivity, and absence of validated quantification. ^68^Ga-NODAGA-RGD can be produced locally in centers with access to a ^68^Ga generator [15] and radiolabeling can be easily done in kit-based or automated modules. It targets the α_v_β_3_ integrins [8–10], and showed promising results in animal trials and demonstrated safe dosimetry profile [16–18]. Patients with different tumor types have also been reported using ^68^Ga-NODAGA-RGD [16, 19, 20], but no specific study has been performed in a HNSCC population.

We aimed at evaluating the potential of ^68^Ga-NODAGA-RGD PET/CT for imaging angiogenesis in HNSCC in comparison to the standard ^18^F-FDG PET/CT regarding tumoral uptake and distribution, as well as histological differentiation.

## Materials and methods

### Study population

Ten consecutive patients were prospectively enrolled with untreated HNSCC of the oral cavity, hypopharynx, or rhinopharynx proven by histology. They were referred by the Department of Head and Neck Surgery to the Department of Nuclear Medicine and Molecular Imaging for a ^18^F-FDG PET/CT. Written informed consent was obtained from study participants. Ethics committee approval was obtained for the protocol (Ethics Commission Vaud, protocol CER-VD #120/12) and from the Swiss national regulatory authorities. The inclusion criteria were age ≤ 85 years, Karnofsky index ≥ 80%, biopsy-proven HNSCC, and signed consent form; exclusion criteria were pregnancy, breastfeeding, and age < 18 years. The biopsy was performed at least 2 weeks before PET/CT imaging.

### Image acquisitions

Both ^18^F-FDG and ^68^Ga-NODAGA-RGD PET/CT were performed at our hospital. Pregnancy test was done before the scan in women of childbearing age before each PET/CT. Patients were asked to fast > 6 h before tracer injection and blood glucose was < 8.3 mmol/L before tracer injection. Vertex to mid-thigh acquisition (8 bed positions, 2 min per bed position, with dedicated 2 bed position acquisition of 3 min per bed position on ear, nose, and throat (ENT) region [vertex to pulmonary apex]) was performed (Discovery 690 TOF, GE Healthcare, Waukesha, WI, USA). ^68^Ga-NODAGA-RGD PET/CT images were acquired 70 min after intravenous administration of 200 MBq ^68^Ga-NODAGA-RGD in an antecubital vein followed by 10 mL of 0.9% NaCl solution, and ^18^F-FDG images were acquired 70 min after intravenous injection of 3.5 MBq/kg ^18^F-FDG in an antecubital vein followed by 10 mL of 0.9% NaCl solution. PET data were reconstructed using OSEM (3 iterations, 16 subsets). Head to mid-thigh unenhanced CT was acquired for attenuation correction (120 kV, 60 mA, 0.8 s/rotation, pitch 0.9, CTDI 4.54 mGy). The mean delay between both PET/CT scans was ≤ 7 days.

### Image analysis

**Fig. 1:**
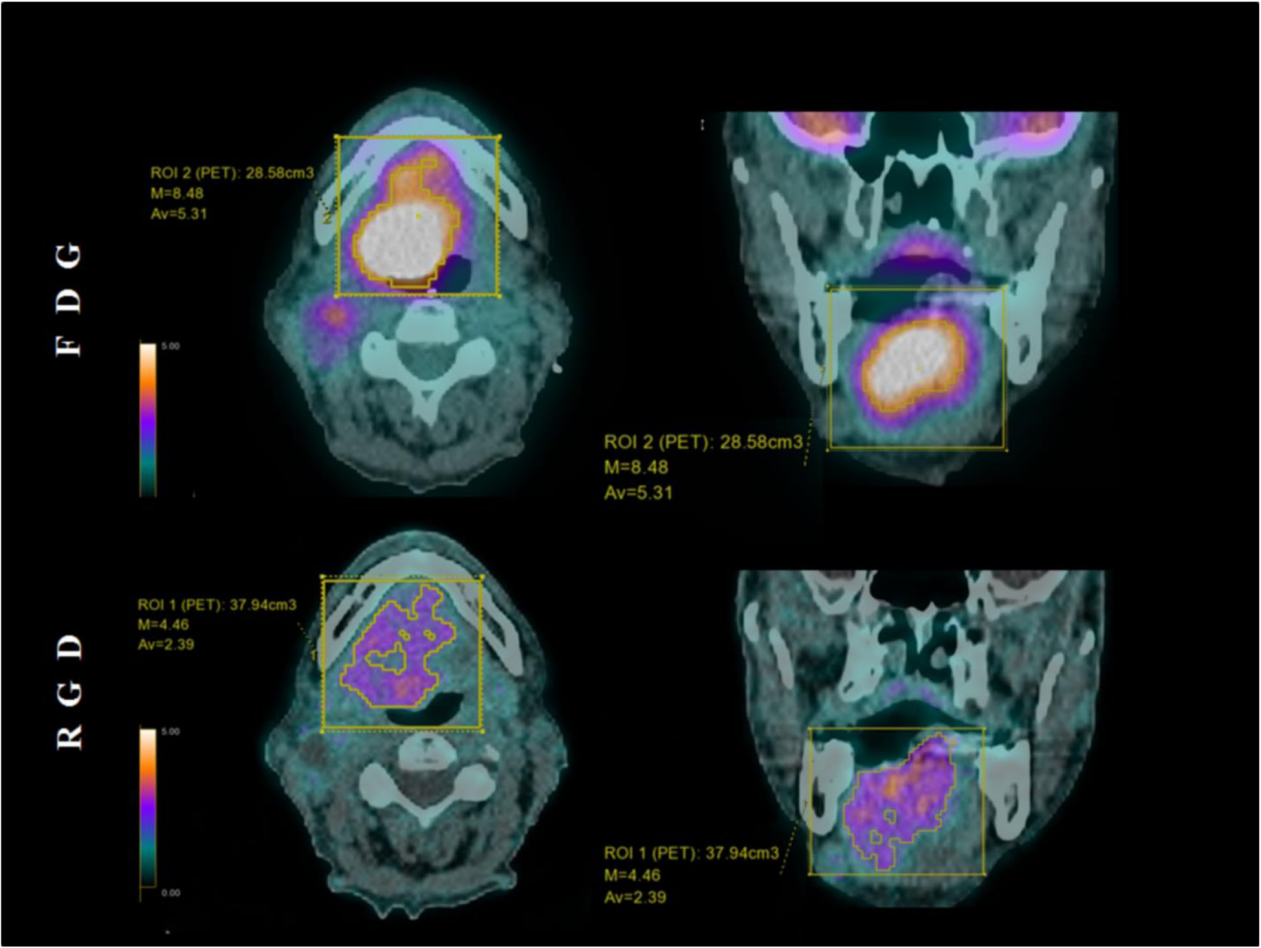
Example of axial and coronal ^18^F-FDG and ^68^Ga-NODAGA-RGD PET/CT 3-D volume of interest semi-automatically delineated on a 42%  SUV_max_ threshold for patient #8 using a parallelepipedal bounding box

Images were post-processed on an Advantage Workstation 4.6 (GE Healthcare, Waukesha, Wisconsin, USA) using multiplanar reformatted images of PET alone, CT alone, and fused PET/CT with linked cursors. Image analysis was performed by two nuclear physicians with respectively 3- and 15-year experience in PET/CT. First, tracers’ distribution was assessed by activity seen in normal anatomical structures and by measuring the maximum and mean SUV (SUV_max_ and SUV_mean_) in the brain, parotid glands, thyroid, mediastinum, myocardium, lung, liver, spleen, colon, small intestine, kidneys, bladder, psoas muscle, and bone marrow (i.e., first lumbar vertebra) from a 42% SUV_max_ thresholded volume of interest (VOI) embedding each structure. Tracers’ uptake was then observed in the primary tumors, lymph nodes, and distant metastases, as well as in any non-tumoral pathological structure. When available, magnetic resonance images were compared to PET images for precise localization of intra-tumoral uptake. SUV_max_ and SUV_mean_ of the primary tumors, lymph nodes, and metastases were semi-automatically extracted from a 3-D volume of interest (VOI) delineated around the lesion using 42% SUV_max_ threshold, as illustrated in Fig. 1. Background uptake was measured in the posterior cervical muscles with a VOI of 1.5 cm^3^ to compute the lesion-to-background ratio. Tracer avid tumor volume (TATV) is the volume within a boundary determined with a 42%  SUV_max_ threshold for ^68^Ga-NODAGA-RGD. For ^18^F-FDG PET, this same 42%  SUV_max_ threshold corresponds to usual metabolic tumor volume (MTV). The size of the lymph node was measured in its short axis.

### Histopathological analysis

All histopathological biopsies were performed in the Department of Head and Neck Surgery and analyses in the Institute of Pathology by a pathologist specialized in head and neck cancers. The analysis of samples included standard histopathology analysis with evaluation of the tumor grade, as well as an immunostaining analysis of p16 and in situ hybridization to detect high-risk HPV.

### Statistical analysis

Continuous variables are presented as mean ± standard deviation (SD). SUV values were compared with the Wilcoxon signed-rank test for differences between ^68^Ga-NODAGA-RGD and ^18^F-FDG scans, as well as for the effect of tumor grade, HPV, and p16 status. The relation between ^68^Ga-NODAGA-RGD and ^18^F-FDG values was assessed using the Spearman correlation coefficient, which was also used to assess the relation between tracers’ uptake and age or lymph node size. Statistics were performed with the Stata 15.1 software (StataCorp, College Station, TX, USA). A *p* value < 0.05 was considered as statistically significant.

## Results

### Study population

We included 10 patients (6 males and 4 females), all Caucasian with a mean age of 58.4 ± 8.3 years (range, 44–73 years). All patients had a proven head and neck carcinoma, with one patient (#5) having two synchronous tumors and one patient (#10) having a dedifferentiated carcinoma (Table 1). Histologic grading showed only 2 patients with poorly differentiated tumor, 4 were well differentiated, and 5 had a moderate differentiation. Finally, one patient (#5) had distant metastases (2 lung lesions).

**Table 1:** Study population

	Gender	Age	TNM	Tumor localization	Biopsy result	Histologic grading	p16	HPV
1	F	63	pT2 pN1 cM0	Left tonsil	SCC	Poorly differentiated	+	+
2	M	59	pT2 pN1 cM0	Left tonsil	SCC	Well differentiated	–	–
3	M	53	pT2 pN2b cM0	Left paramandibular	SCC	Moderate differentiated	+	+
4	M	50	pT3 pN0 cM0	Left arytenoid	SCC	Well differentiated	+	+
5	F	73	pT1b pN2b cM1	Glottic	SCC	Well differentiated	–	–
	F	73	pT3 pN2b pM1	Posterior oral cavity	SCC	Well differentiated	–	–
6	F	65	pT4a pN2c cM0	Right tongue base	SCC	Moderate differentiated	+	+
7	M	49	cT2 cN3b cM0	Left tonsil	SCC	Moderate differentiated	–	–
8	F	69	pT4a pN2c cM0	Base of the tongue	SCC	Moderate differentiated	–	–
9	M	59	pT3 pN3 cM0	Left tonsil	SCC	Moderate differentiated	–	–
10	M	44	pT2 pN2 cM0	Right rhinopharynx	Dedifferentiated carcinoma	Poorly differentiated	+	+

### PET/CT imaging

PET/CT images were acquired 71 ± 14 min (range, 56–90 min) after administration of 216 ± 79 MBq (range, 208–250 MBq) ^68^Ga-NODAGA-RGD. For ^18^F-FDG, images were acquired 70 ± 11.5 min (range, 63–93 min) after injection of 3.5 MBq/kg (range, 185–291 MBq). The mean time elapsed since ^18^F-FDG and ^68^Ga-NODAGA-RGD PET/CT scans was 2.5 ± 1.8 days (range, 1–7 days). Both radiopharmaceuticals were well-tolerated, and no radiopharmaceutical-related adverse effect was observed. The mean time elapsed since biopsy and PET/CT imaging was 17.5 ± 5.3 days (range, 14–24 days).

### ^68^Ga-NODAGA-RGD distribution

**Fig. 2:**
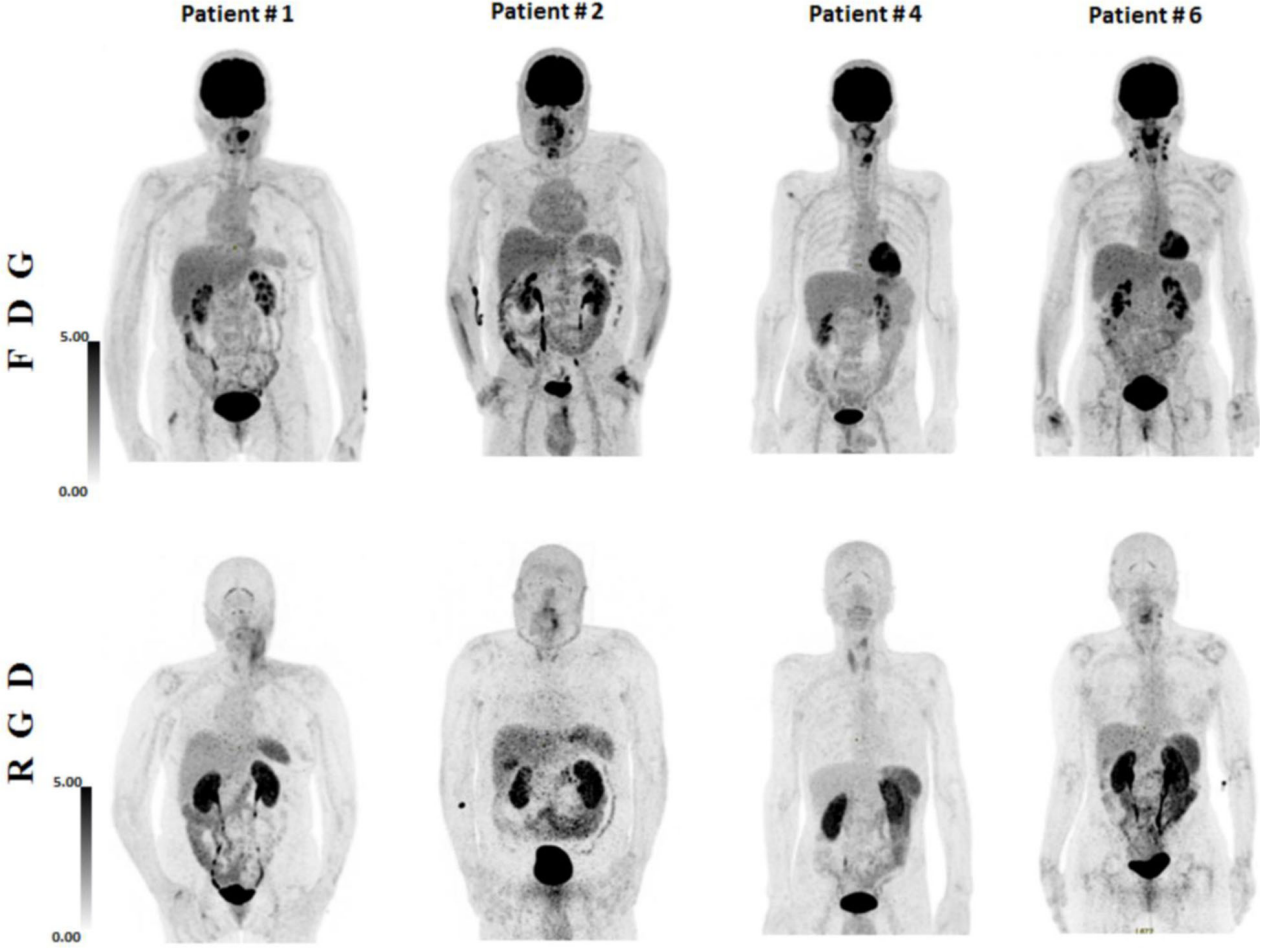
Maximum intensity projection (MIP) of ^18^F-FDG and ^68^Ga-NODAGA-RGD PET/CT in four patients. HNSCC primary tumor had a significant uptake in all patients. Lymph nodes also demonstrated a significant uptake as seen in patients #1 and #6. A focal uptake is detectable with ^68^Ga-NODAGA-RGD PET/CT in the liver of patient #2, corresponding to the gallbladder. Inflammatory capsulitis of the glenohumeral joint was also observed in patients #1 and #6

**Fig. 3:**
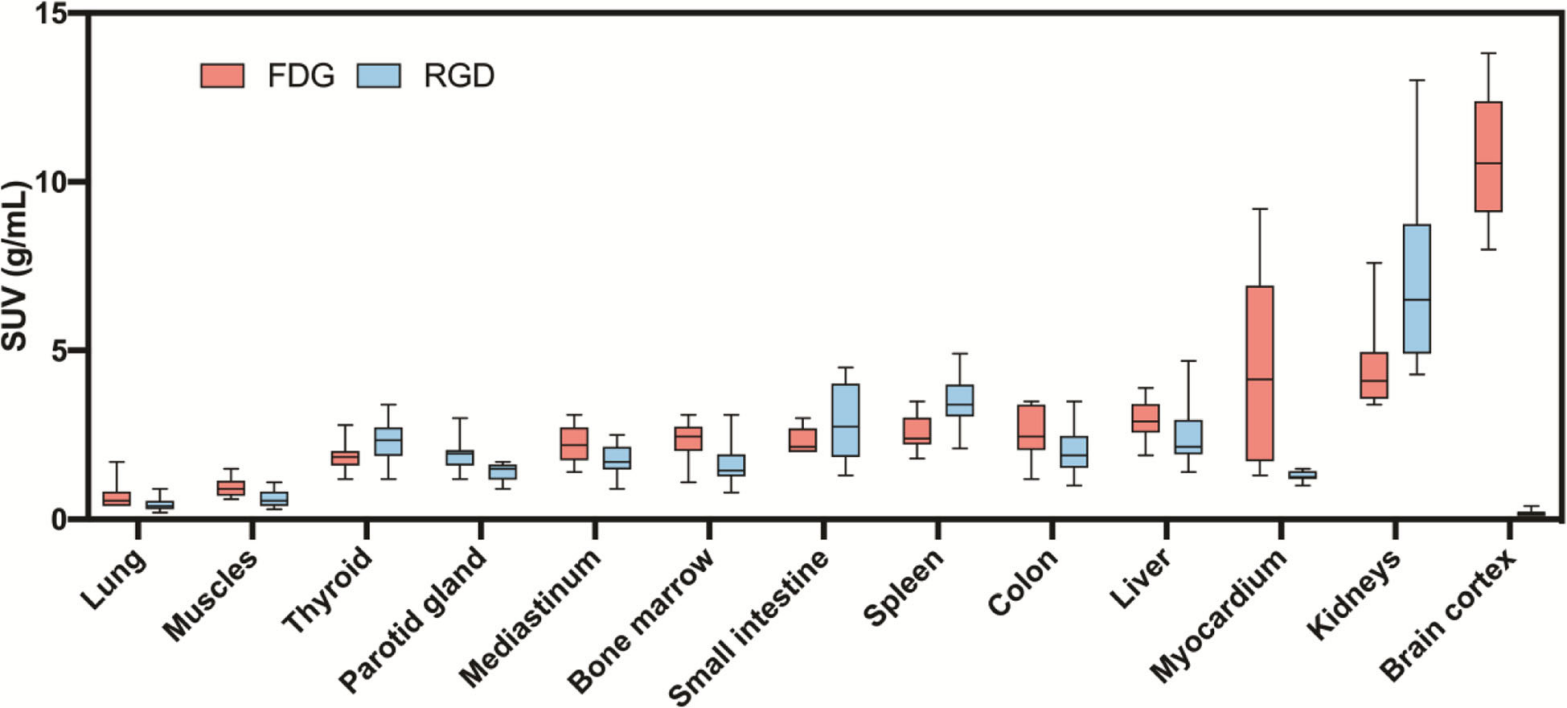
Box plot **c**omparison of ^18^F-FDG and ^68^Ga-NODAGA-RGD SUV_max_ in organs. SUV_max_ was significantly different between both tracers in all organs (all *p* < 0.037), except in the thyroid, the gut (small intestine and colon), and the bladder (not shown) (all *p* > 0.10). The most significant difference was observed for the brain and the myocardium, which presented only minimal ^68^Ga-NODAGA-RGD uptake compared to ^18^F-FDG

^68^Ga-NODAGA-RGD in comparison to ^18^F-FDG PET/CT images demonstrated different whole-body distributions in all the ten patients. Figure 2 displays body tracers’ distribution of four selected patients. Compared to ^18^F-FDG images, ^68^Ga-NODAGA-RGD images demonstrated significantly higher uptake in the spleen and in the kidneys, while the uptake was lower in the brain, the parotid glands, the mediastinum, the myocardium, the lung, the liver, the psoas muscle, and the bone (all *p* < 0.037, Fig. 3). Similar uptake was measured in the thyroid gland, the gut, and the bladder (all *p* > 0.1).

Non-tumoral positive uptake regions were seen in several patients for both tracers, notably due to inflammatory diseases. The majority of them were seen in patients #1, #4, #5, and #6 and were analyzed as glenohumeral joint inflammation proven by clinical data. In patients #1, #2, and #6, stomatitis was proven by mouth and throat examination.

### Analysis in the primary tumors

**Fig. 4:**
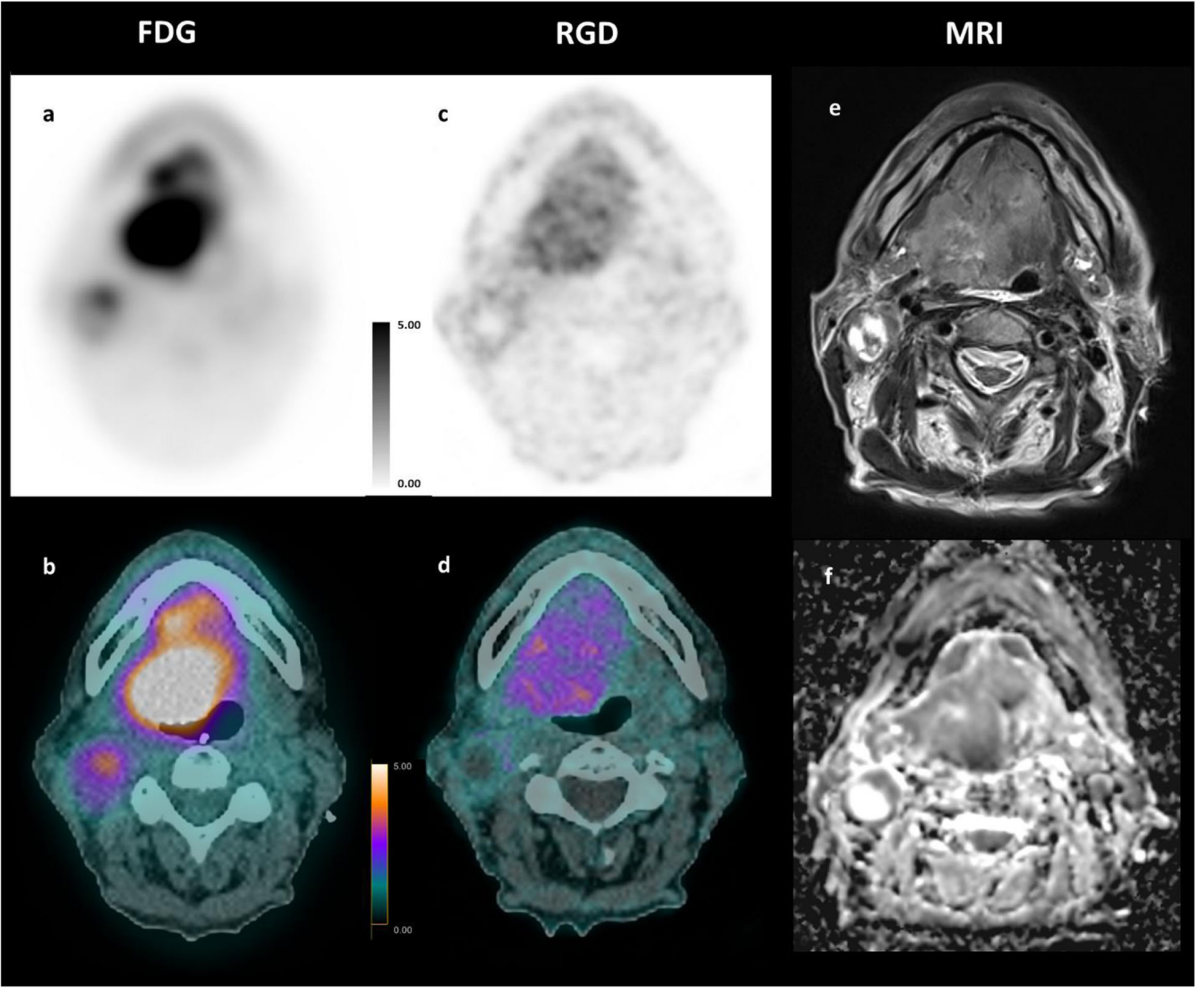
Comparative MRI, ^18^F-FDG, and ^68^Ga-NODAGA-RGD PET/CT of patient #8. Axial PET/CT fusion slices of a 69-year-old man with a moderate differentiated base tong SCC. The images show different tumor-to-background ratios in between the two radiotracers ^18^F-FDG PET/CT (**a**, **b**) vs. ^68^Ga-NODAGA-RGD PET/CT (**c**, **d)**, and also a slightly different distribution of activity within the tumor bed when compared with the MR images **e** T2w and **f** ADC map of diffusion

**Fig. 5:**
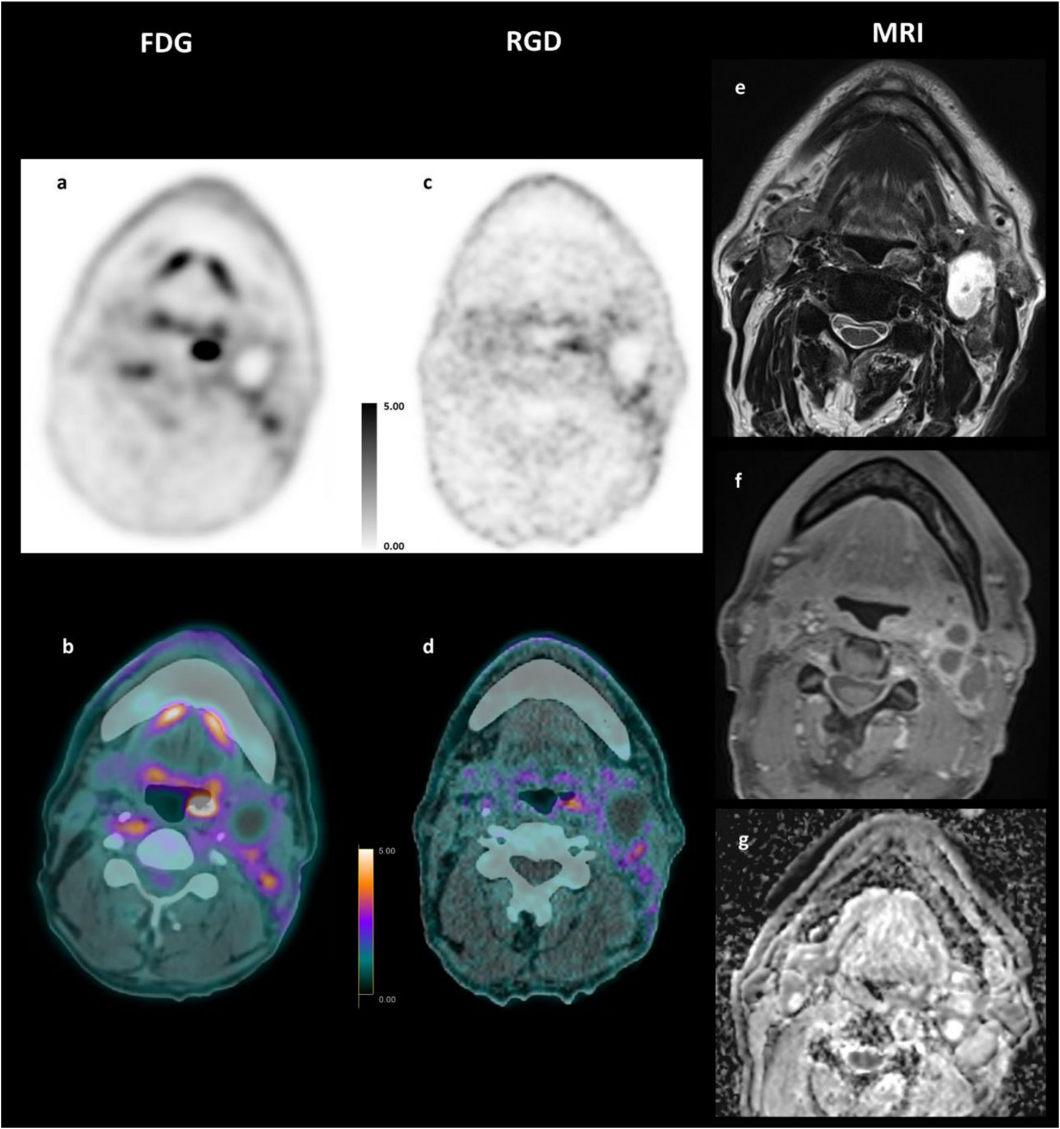
Comparative ^18^F-FDG (**a**, **b**), ^68^Ga-NODAGA-RGD PET/CT (**c**, **d**), and MRI (**e** T2w axial, **f** T1w post Gadolinium, **g** ADC map of diffusion), of patient #9 (59-year man with moderate differentiated left tonsil squamous cell carcinoma). The ^18^F-FDG and ^68^Ga-NODAGA-RGD PET images showed different signal-to-noise ratios and a slightly different distribution of activity within the tumor bed. The left cervical lymph node showed a photopenic center with absence of tracer uptake for both tracers corresponding to necrosis on MRI

All primary tumors were visually detectable with both tracers (Table 2). Distribution of the tracers within the tumors was different as shown on the axial PET/CT fusion (Fig. 1). Compared to magnetic resonance images for tumor delineation, we noticed that ^18^F-FDG uptake was mostly homogenous inside the tumors. ^68^Ga-NODAGA-RGD PET showed heterogenous uptake within the tumors. In patient #8 (Fig. 4) for instance, moderate uptakes were seen mostly in the periphery of the tumor. Necrotic areas did not display significant uptake for both tracers (Fig. 5).

**Table 2:** SUV and TATV results of primary tumors

Patient	SUVmax [g/mL]		SUVmean [g/mL]		SUV42%/SUVbackground [1]		Tracer avid tumor volume [cm^3^]	
	FDG	RGD	FDG	RGD	FDG	RGD	FDG	RGD
1	11.6	3.5	9.1	2.4	9.3	3.3	3.7	21
2	14.3	2.5	12.5	2.9	15.3	5	2.3	12.9
3	20.6	5.2	9	2.1	16.6	3.8	10.5	34.7
4	10.6	3.2	6.7	1.9	10.8	2.7	2.4	8.1
5	7.6	3.1	5.5	0.4	10.8	2.8	1.2	3.5
	9.7	4.8	8.7	2.7	8.7	4.4	4.2	12.3
6	16.6	4.4	10.4	2.8	9.1	4.8	8.5	17
7	8.6	4.8	5	2.7	9.5	5.3	5	6.4
8	8.5	4.5	5.3	2.3	7.7	4.8	28.6	38
9	8.7	2.1	4.9	0.9	9.6	2	6	8.1
10	28.3	2.3	14	1.2	31.4	2.5	10	16
Mean	14	3.9	8.2	2.0	12.7	3.8	9.6	14
SD	6.1	1.1	3.1	0.8	4.6	1.0	11.2	9.4
*p*		0.0017		0.0017		0.0017		0.085

**Fig. 6:**
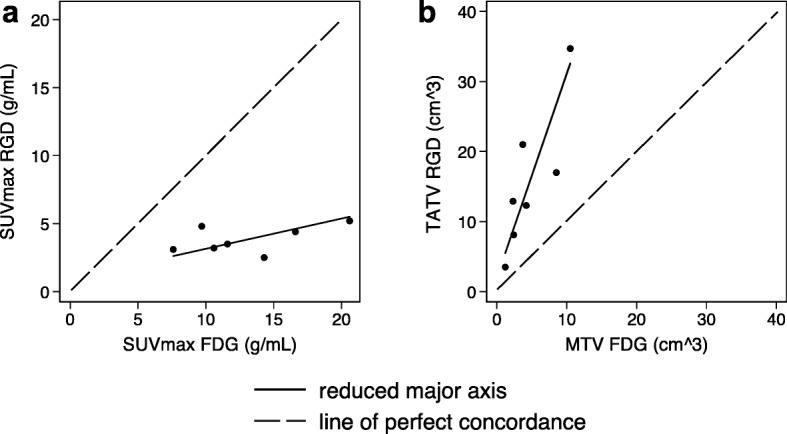
Correlation between **a**^18^F-FDG and ^68^Ga-NODAGA-RGD SUV_max_, which was systematically lower (slope of the reduced major axis 0.22 < 1.00) and **b**^68^Ga-NODAGA-RGD tracer avid tumor volume (TATV), which was systematically higher than ^18^F-FDG metabolic tumor volume (MTV) (slope of the reduced major axis 2.91 > 1.00)

Tumor uptake was significantly higher with ^18^F-FDG than with ^68^Ga-NODAGA-RGD (SUV_max_ 14.0 ± 6.1 g/mL versus 3.9 ± 1.1 g/mL, *p* = 0.0017; SUV_mean_ (8.2 ± 3.1 g/mL versus 2.0 ± 0.8 g/mL, *p* = 0.0017) as was the tumor-to-background ratio (Table 2). One patient showed very low ^68^Ga-NODAGA-RGD activity (patient #9). A linear positive correlation between the ^18^F-FDG and the ^68^Ga-NODAGA-RGD SUV_mean_ was found (Spearman’s rho = 0.89, *p* = 0.0068), but not for SUV_max_ values (Spearman’s rho = 0.39, *p* = 0.38). There was no statistically significant relation between age and tracers’ uptake (*p* = 0.5). As seen in Table 2, “tracer avid tumor volume” was larger with ^68^Ga-NODAGA-RGD PET/CT with a volume around 30% higher for ^68^Ga-NODAGA-RGD (Fig. 6), but this difference did not reach statistical significance (*p* = 0.085) in comparison to ^18^F-FDG PET/CT. There was no significant correlation between the uptake volumes of the two tracers (Spearman’s rho = 0.038, *p* < 0.05).

### Analysis in the lymph nodes and metastases

All lymph nodes and distant metastases were seen with both tracers. In some cases, such as in patients #9 and #10, ^68^Ga-NODAGA-RGD uptake was however very low, with a target-to-background ratio < 2 (Table 3). The size of the lymph node was measured in short axis (8.5 ± 2.7 mm; range, 4–15 mm), and there was no significant correlation between lymph node size and uptake (*p* > 0.05). Tracer avid tumor volume was always higher with the ^68^Ga-NODAGA-RGD PET in the lymph nodes, as seen with in the primary tumors.

**Table 3:** SUV and TATV results of lymph nodes and distant metastases

Patient	Localization	Dimension^a^ [mm]	SUVmax [g/mL]		SUVmean [g/mL]		SUV42%/SUVbackground [1]		Tracer avid tumor volume [cm^3^]	
			FDG	RGD	FDG	RGD	FDG	RGD	FDG	RGD
1	Left IIb	10	3	3.2	1.9	2	3.3	4.5	3.7	5
2	Left IIa	9	4.7	1.3	2.6	0.8	4.3	2.2	3.5	4.4
3	Left IIa–IIb	4	15	3.1	8.4	1.8	16.6	3.9	8.5	14
4	None	–	–	–	–		–	–	–	–
5	Right IIa	10	11.2	2.8	6.7	1.8	14	3.5	1	5.4
	Right IIa	9	13	4.8	9	2.9	16.2	6	3.2	7.5
	Right IIa	6	3.9	3.5	2.3	2.1	4.9	4.4	2.4	5
6	Left IIa	8	7.7	3.5	4.5	2	8.5	4.4	1.3	2.5
	Right III	15	6	2.2	3.6	1.2	6.7	2.8	1.4	4.7
	Left III	9	5.2	2.5	3	1.4	5.8	3.1	1.6	4.1
	Right IV	7	6	1.9	3.7	1.1	6.7	2.4	1.5	5.2
	Left IV	7	7.8	1.9	4.6	1.2	8.7	2.4	1.1	4.5
	Right V	7.5	7.4	1.7	4.8	1	8.2	2.1	1	4.5
7	Right IIa	9	4	1.9	2.2	1.1	4.4	2.4	3	5
	Left IIb	5	2.3	2.4	1.3	1.3	2.5	3	3.2	4.6
8	Right IIa	14	4	1.2	2.5	0.9	4.4	1.5	4	7
	Right IIb	13	7	2.4	4.1	1.4	7.8	3	3.6	6
9	Left I	17	4	1.3	2.1	1	4.4	1.6	5.7	8.2
	Left IIa	17	4.5	1.4	2.4	1	5	1.7	6	9
	Left IIb	12	4	1.4	2.1	0.9	4.4	1.7	4.5	5.9
	Left III	13	5	2.2	3	1.2	5.5	2.7	4	6.5
10	Left retropharyngeal	7	7	1.4	4.1	1	7.8	1.7	5.6	8
	Right retropharyngeal	5	4	1	2	0.8	4.4	1.2	3	5.3
	Right IIa	7	7	1.5	4	1.3	7.8	1.9	5	7.7
Mean		8.5	7	2.5	3.5	1.3	6.8	2.8	3.2	6.1
SD		2.7	4.1	1.2	2.6	0.7	5	1.5	2.1	5.7
*p*				0.0017		0.0017		0.0017		0.0017
**5**	Pulmonary right upper lobe	16	12	2.3	7.7	1.4	15	2.9	0.9	2
	Pulmonary left upper lobe	10	11.8	1.2	7.7	0.7	14.8	1.5	0.7	3.6
Mean			11.9	1.7	7.7	0.8	14.9	2.2	0.8	2.8

^a^Lymph node measure: small axis

Metastatic spread of the disease was seen only in patient #5, with bilateral lung metastases. Lower SUV_max_ was reported with the ^68^Ga-NODAGA-RGD PET (1.7 g/mL versus 11.9 g/mL) and higher tracer avid tumor volume (2.8 mL versus 0.8 mL). No statistical analysis of metastatic disease was performed because of the paucity of lesions.

### Effect of tumor grade, p16, and HPV status

Both radiotracers’ uptakes did not correlate with tumor grade (*p* ≥ 0.17). P16 and HPV immunostaining showed a good association between the p16 and HPV tests (*p* < 0.05). Five histopathological analyses were HPV and p16 positive and six were negative. Mean SUV_max_ values of p16 and HPV positive cases were 16.4 ± 6.9 g/mL with ^18^F-FDG and 3.8 ± 1.0 g/mL with ^68^Ga-NODAGA-RGD. Mean SUV_max_ values of p16 and HPV negative cases were respectively of 9.8 ± 1.7 g/mL with ^18^F-FDG and 4.1 ± 1.2 g/mL with ^68^Ga-NODAGA-RGD. No significant difference in both tracers’ uptake was found regarding HPV or p16 protein expression (*p* = 0.22) (Table 4).

**Table 4:** SUV_max_ of HPV—p16 positive and negative cases

	HPV and p16 positive		HPV and p16 negative	
	FDG	RGD	FDG	RGD
	14.3	4.3	10.6	5.4
	15.7	4.2	9.7	3.1
	10.6	3.2	13	4.8
	13	4.8	8.6	4.8
	28.3	2.3	8.5	4.5
	-		8.7	2.1
Mean	16.4	3.8	9.8	4.1
SD	6.9	1.0	1.7	1.2
*p*	0.22		0.22	

## Discussion

Our pilot study is the first study on humans to systematically compare ^18^F-FDG and ^68^Ga-NODAGA-RGD uptake in a HNSCC patient population. It shows that: (1) every primary HNSCC tumor and lymph nodes were visually detectable with both tracers, but with different uptake patterns; (2) ^68^Ga-NODAGA-RGD uptake was heterogeneous with a low target-to-background ratio while ^18^F-FDG uptake is mostly homogeneous with higher target-to-background ratio; and (3) ^68^Ga-NODAGA-RGD uptake was not related to tumor grade, p16, or HPV status.

^18^F-FDG PET-CT has a high clinical value in the initial workup and follow-up of patients with HNSCC tumors [4–7]. It however only allows evaluation of tumor cell metabolism but not neoangiogenesis. To this purpose, we conducted a one-to-one comparison of tracers to assess the clinical potential of ^68^Ga-NODAGA-RGD. All HNSCC primary tumors, lymph nodes, and metastases detected on ^18^F-FDG PET/CT images were also seen with the angiogenesis radiotracer. Only few studies have been conducted in humans; while Haubner et al. [20] demonstrated that ^68^Ga-NODAGA-RGD uptake was not sufficient to be used in patients with hepatocellular carcinoma, other authors reported sufficient uptake for diagnostic purpose in human xenografts of esophageal carcinoma, melanoma, and glioblastoma [18, 21]. As both ^68^Ga-NODAGA-RGD and ^18^F-Galacto-RGD demonstrated similar preclinical results [22], our results are in line with the previous work by Beer et al. [8], who concluded that thanks to its significant uptake, ^18^F-Galacto-RGD might be used for the assessment of angiogenesis and for planning and response evaluation of α_v_β_3_ targeted therapies in HNSCC.

However, it is worth to mention that tracer uptake patterns were very different between ^18^F-FDG and ^68^Ga-NODAGA-RGD. Indeed, TATV was larger with ^68^Ga-NODAGA-RGD, with heterogeneous uptake within the primary tumor and lymph nodes, and relative low target-to-background ratio compared with ^18^F-FDG. While this seems to preclude the use of ^68^Ga-NODAGA-RGD as a single tracer for tumor staging, we assume that it brings complementary information about the tumor itself. Part of volume difference can be due to difference in positron energy between the fluorine-18 and gallium-68. Also, the threshold used for TATV delineation is subject to discussion. We used a 42% SUV_max_ fixed threshold similarly to MTV delineation, which may have resulted in larger TATV due to lower SUV_max_ values with ^68^Ga-NODAGA-RGD. Threshold adaptation for ^68^Ga-NODAGA-RGD could be performed and defined based on tumor margins if defined on whole tumor histopathological specimen, which was out of the scope of our study, as not all tumors and lymph nodes were resected in toto. Nevertheless, we believe that difference in uptake patterns and volume are mainly attributable to difference in the tracer targeting. Although we did not perform the immunohistochemistry staining of human HNSCC tissue microarray to properly correlate uptake with angiogenesis [23], it is known that ^68^Ga-NODAGA-RGD improves imaging of α_v_β_3_ expression [24]. Beer et al. [8] demonstrated that the uptake of ^18^F-Galacto-RGD mostly represented α_v_β_3_ expression in the neovasculature, but not in the HNSCC tumor cells themselves. This was also confirmed with other RGD-based tracers on HNSCC tumor xenografts [25]. ^68^Ga-NODAGA-RGD uptake beyond ^18^F-FDG avid areas could thus reflect the presence of the formation of neovessels. Isal et al. [21] demonstrated that tumor areas with high ^68^Ga-NODAGA-RGD uptake also exhibited the highest rates of cell proliferation and integrin expressions irrespective of cell density in engrafted glioblastomas. This seems to be different in HNSCC, as we did not find any significant association between tracers’ uptake and HNSCC grade. Despite different uptake patterns, we found a significant correlation between ^18^F-FDG and ^68^Ga-NODAGA-RGD SUV_mean_ values, which overall might indicate the coexistence of interrelated pathophysiological phenomenon within the tumor, i.e., cell proliferation and neoangiogenesis. Finally, no significant difference in ^68^Ga-NODAGA-RGD activity was found regarding HPV or p16 protein expression (*p* ≥ 0.22). Although HPV and p16 have demonstrated significant prognostic value in HNSCC tumors [1, 26], this may not preclude the use of ^68^Ga-NODAGA-RGD as a prognostic biomarker. Indeed, taking into account that tumor neovessels are of paramount importance for tumor oxygenation, the prognostic value of ^68^Ga-NODAGA-RGD could be assessed in HNSCC patients undergoing chemoradiotherapy. Recent preclinical [27] and clinical pilot studies [28] hence reported that ^111^In-RGD2 and ^18^F-RGD-K5, two tracers targeting integrin α_v_β_3_, having the potential to monitor response to therapy and to identify patients with incomplete responses to concurrent chemoradiotherapy. This point has to be explored in a larger prospective study.

We acknowledge several limitations inherent to a pilot study. First, we evaluated a small sample of HNSCC patients, which limits discriminating power, especially regarding correlation with histology. Second, immunostaining was not performed to confirm regional α_v_β_3_ expression, but rather characterize whole tumor distribution. Third, as already mentioned, we used a fixed threshold for TATV definition in a first approximation, which might have overestimated tumor volume; threshold optimization based on spatial comparison with α_v_β_3_ immunostaining on whole-tumor histological slices would need to be performed for more precision. Finally, larger, longitudinal studies would need to be performed to determine the prognostic value of ^68^Ga-NODAGA-RGD.

## Conclusion

Our study revealed that HNSCC primary tumors, lymph nodes, and pulmonary metastases can be visualized with both ^18^F-FDG and ^68^Ga-NODAGA-RGD PET/CT. While SUV_mean_ values were correlated among both tracers, intensities were largely different and were not influenced by HPV or p16 status. This indicates potential complementary use of both tracers. Further studies are now needed to elucidate the respective role of ^68^Ga-NODAGA-RGD in the workup of patients with HNSCC.

## Data Availability

The datasets used and/or analyzed during the current study are available from the corresponding author on reasonable request.
